# Mycosynthesis of silver nanoparticles from endophytic *Aspergillus flavipes* AUMC 15772: ovat-statistical optimization, characterization and biological activities

**DOI:** 10.1186/s12934-023-02238-4

**Published:** 2023-11-06

**Authors:** Nessma A. EL-Zawawy, Alaa M. Abou-Zeid, Doha M. Beltagy, Nada H. Hantera, Hoda S. Nouh

**Affiliations:** 1 Botany Department, Faculty of Science, Tanta University, Tanta, Egypt; 2 Biochemistry Department, Faculty of Science, Damanhour University, Damanhour, Egypt

**Keywords:** Mycosynthesis of SNPs, Endophytic fungi, Optimization, FTIR, X-ray, XRD, TEM, Antimicrobial activity

## Abstract

**Background:**

Mycosynthesis of silver nanoparticles (SNPs) offers a safe, eco-friendly, and promising alternative technique for large-scale manufacturing. Our study might be the first report that uses mycelial filtrate of an endophytic fungus, *Aspergillus flavipes*, for SNPs production under optimal conditions as an antimicrobial agent against clinical multidrug-resistant (MDR) wound pathogens.

**Results:**

In the present study, among four different endophytic fungi isolated from leaves of *Lycium shawii*, the only one isolate that has the ability to mycosynthesize SNPs has been identified for the first time as* Aspergillus flavipes* AUMC 15772 and deposited in Genebank under the accession number OP521771. One variable at a time (OVAT) and Plackett Burman design (PBD) were conducted for enhancing the production of mycosynthesized SNPs (Myco-SNPs) through optimization using five independent variables. The overall optimal variables for increasing the mycosynthesis of SNPs from mycelial filtrate of* A. flavipes* as a novel endophytic fungus were a silver nitrate concentration of 2 mM, a pH of 7.0, an incubation time of 5 days, and a mycelial filtrate concentration of 30% in dark conditions. UV–visible spectroscopy (UV–Vis), Fourier transform infrared spectroscopy (FT-IR), X-ray spectroscopy (XRD), Transmission electron microscopy (TEM), and Selected-Area Electron Diffraction (SAED) patterns were used to characterize Myco-SNPs, which showed the peak of absorbance at 420 nm, and FTIR showed the bands at 3426.44, 2923.30, 1681.85, 1552.64, and 1023.02 cm-1, respectively, which illustrated the presence of polyphenols, hydroxyl, alkene, nitro compounds, and aliphatic amines, respectively. The XRD pattern revealed the formation of Myco-SNPs with good crystal quality at 2θ = 34.23° and 38.18°. The TEM image and SAED pattern show the spherical crystalline shape of Myco-SNPs with an average size of 6.9232 nm. High antibacterial activity of Myco-SNPs was recorded against MDR wound pathogens as studied by minimum inhibitory concentrations ranging from 8 to 32 µg/mL, time kill kinetics, and post-agent effects. Also, in vitro cell tests indicated that Myco-SNPs support the cell viability of human skin fibroblast cells as a nontoxic compound.

**Conclusion:**

The obtained results revealed the successful production of Myco-SNPs using the mycelial filtrate of *A. flavipes,* which may be a promising nontoxic alternative candidate for combating MDR wound pathogens.

## Introduction

Nanotechnology is considered one of the most important emerging fields in material science for the purpose of applications and the synthesis of nanomaterials [1]. At the nanoscale level, materials have a scale size between 1 and 100 nm with different chemical, physical, and magnetic properties [2]. These unique properties allow them to interact with different microorganisms [3]. There are many potential applications for metal nanoparticles in biomedicine, antimicrobial activity, optics, and catalysis [4], especially SNPs, which have gained special attention due to their effectiveness in medical fields as therapeutics [5, 6] and in drug delivery [7].

The biosynthesis of SNPs is a priority area of research in nanotechnology [8]. Advantages of biosynthesis of SNPs are reduced toxicity levels, cost, and time compared to chemical and physical methods [9]. Different microorganisms, such as fungi, bacteria, and yeast, can produce SNPs through extracellular or intracellular pathways [1]. Fungi can produce large amounts of proteins that help increase the productivity of SNPs [10]. Although extracellular mycosynthesis of SNPs has been reported using many molds such as *Aspergillus niger* and *Penicillium sp.* [11], there aren’t enough reports on the mycosynthesis of SNPs by endophytic fungi, which colonize the internal tissues of plants in a mutualistic relationship and are considered a potential source of bioactive compounds [12].

Optimizing the factors affecting the biosynthesis of SNPs is an important step for enhancing the production of SNPs and controlling the biosynthesis process. These factors are temperature, pH, inoculum size, and metal ion concentration [13]. Fractional factorial designs are sets of statistical tools such as the Plackett–Burman Design (PBD), which is very effective in determining the most important variables affecting the biosynthesis process [14]. Nowadays, these designs have been used in the optimization of several bioprocesses [15].

Microbial infections in wounds are a significant cause of mortality and morbidity [12]. The most pathogenic MDR microorganisms isolated from infected wounds are* Staphylococcus aureus* [16],* Pseudomonas aeruginosa* [17], *Escherichia coli* [18], and *Klebsiella pneumonia* [19]. Multidrug resistance is considered an important challenge in treating such pathogens [20]. Until now, effective treatment of wound infections against MDR pathogens has been inadequate, making alternative approaches for novel antimicrobial agents highly desirable.

To the best of our knowledge, this study is the first to describe the optimization and characterization of the extracellular mycosynthesis of SNPs from novel endophytic fungi (*A. flavipes,* AUMC 15772). Moreover, this work will investigate the potential of SNPs as a promising, safe antimicrobial agent against MDR wound pathogens that may open up a new avenue for medical and pharmaceutical applications.

## Materials and methods

### Source of fungi and culture maintenance

Four different strains of endophytic fungi coded as F1, F2, F3, and F4 that were previously isolated from the leaves of *Lycium shawii* [15] were cultivated on petri plates containing Sabrouad dextrose agar (SDA) medium supplemented with 100 U/mL penicillin [21]. After incubation for 5–7 days at 30 ℃, all plates were kept at 4 ℃ for further use.

#### Screening of SNPs producing endophytic fungi and mycosynthesis

One milliliter of spore suspension (10^6^ spores/mL) of each endophytic fungus was inoculated separately with 100 mL of sterile Sabrouad broth (SDB) and then incubated at 30 ℃ for 5 days at 200 rpm [22]. After filtration, biomass was washed twice with sterile deionized water. Twenty grams of each fungal mycelium were added to sterile deionized water (100 mL), then incubated at 200 rpm for 2 days at 30 ℃. After filtration, 200 mL of 0.5 M AgNO_3_ solution were added to 100 mL of each mycelial filtrate separately to obtain a final concentration of 1 mM AgNO_3_ and incubated at 30 ℃, 200 rpm in the dark. A visual alteration in color to yellowish brown served as a visual indicator of the reduction of Ag + ions, and the intensity of the reduction was measured spectrophotometrically at 300–500 nm [22]. Silver nitrate solution and uninoculated media were used as controls.

#### Identification of the most potent SNPs producing endophytic fungi

The most potent SNPs producing endophytic fungi were initially identified using macro- and micromorphological traits according to the standard protocols [24, 25]. The light microscope (LM) was used after staining with lactophenol cotton blue stain to examine the hyphal morphology of the selected isolate. Also, scanning electron microscopy (SEM) (JEOL, JSM-5200 LV, Tokyo, Japan) was used to visualize the fungal spores after being coated with gold or palladium (40–60%). Then the selected isolate was forwarded to the Molecular Biology Research Unit, Assiut University, for DNA extraction using the Patho-gene-spin DNA/RNA extraction kit provided by Intron Biotechnology Company, Korea. The fungal DNA was then forwarded to Sol Gent Company, Daejeon, South Korea, for polymerase chain reaction (PCR) and ITS sequencing of the rRNA gene. Two primers, ITS1 (forward) and ITS4 (reverse), were added to the reaction mixture to be used in PCR. Primers have the following composition: ITS1 (5′-TCC GTA GGT GAA CCT GCG G-3′), and ITS4 (5-TCC TCC GCT TAT TGA TAT GC-3′). The purified PCR products were sequenced using the same primers with the incorporation of dideoxy-nucleotides (ddNTPs) in the reaction mixture [26]. The Basic Local Alignment Search Tool (BLAST) available on the National Center for Biotechnology Information (NCBI) website was used to examine the obtained sequences. Meg Align (DNA Star & Baser) software version 5.05 was used to perform phylogenetic analysis of the sequences. After a close match with comparable sequences acquired from the gene bank (using clustal-W method), the phylogenetic tree was viewed and taken as a print screen to be included in the results. Sequences were also sent to GenBank to obtain the accession number.

#### Optimization studies on mycosynthesis of SNPs

To determine the effect of different factors affecting the mycosynthesis of SNPs from selected isolate, two designs of experiments were used as follows: the one variable at a time (OVAT) and the Plackett–Burman fractional factorial design (PBD).

#### One variable at time (OVAT) method

Many environmental factors may have different effects not only on microbial development but also on the reduction of silver ions and yield production [27]. To produce the most rapid and stable Myco-SNPs, AgNO_3_ concentration, pH, temperature, and mycelial filtrate concentration were all tuned. The one variable at a time (OVAT) strategy was employed for optimization by changing one investigative variable at a time while leaving the others unchanged [14].

#### Effect of silver nitrate (AgNO_3_) concentrations

The concentration of the substrate affects the amount of nanoparticles produced. The effect of AgNO_3_ was studied at different concentrations (1, 2, 3, and 4 mM) on the mycosynthesis of SNPs from selected isolate as mentioned previously by Gurunathan et al. [28], with minor changes. The absorbance of the resultant solution was determined using UV–visible absorption spectroscopy after incubation at pH 7, 30 ℃, for 5 days in the dark.

#### Effect of pH

pH has a significant impact on both the production of metabolites and growth, both of which are necessary for the mycosynthesis of SNPs. To determine how pH affected the synthesis of SNPs from the selected isolate, different pH were used at 5, 6, 7, and 8 after incubation of the mycelial filtrate of the selected isolate with AgNO_3_ (1 mM) at 30 ℃ for 5 days in the dark. The absorbance of the resultant mixtures was examined using UV–visible absorption spectroscopy.

#### Effect of temperature

The effect of various temperatures on the mycosynthesis of SNPs from selected isolate was studied at pH 7. Mycelial fungal filtrate of the selected isolate was treated with AgNO_3_ at a concentration of 1 mM and incubated in the dark at (20, 25, 30, and 35 ℃). Then, the absorbance of the resulting mixtures was examined using UV–visible absorption spectroscopy [29, 30].

#### Effect of mycelial filtrate concentrations

Different concentrations of fungal filtrate from selected isolate (10, 20, 30, 40) (V/V) were inoculated into four Erlenmeyer conical flasks, respectively. All flasks were treated with 1 mM AgNO_3_ at pH = 7 and incubated in the dark for 5 days at 30 ℃. Then the absorbance of each flask was measured spectrophotometrically, as previously mentioned [31].

#### Effect of incubation time

The effect of incubation period on mycosynthesis of SNPs from selected isolate was optimized, as mentioned previously, using different time intervals (1, 3, 5, and 7 days). Mycelial filtrate of the selected isolate was treated with AgNO_3_ at a concentration of 1 mM and incubated in the dark at 30 ℃ at pH = 7, then the absorbance of each flask was measured spectrophotometrically [23].

### Plackett Burman design (PBD)

A Plackett–Burman experimental design [32, 33] was applied to determine different variables affecting the mycosynthesis of SNPs from selected isolate. Five independent variables were chosen: AgNO_3_ concentration, medium pH, incubation time, mycelial filtrate concentration, and illumination condition.

These factors were given the designations X_1_, X_2_, X_3_, X_4_, and X_5_, correspondingly, with high (+ 1) and low (−1) levels for each variable as they were chosen based on our preliminary findings (Table 1). In eight trials, five independent variables were arranged using the Plackett–Burman design. All trials were performed in duplicate and incubated at 30 ℃ with shaking at 200 rpm. The mycosynthesis of SNPs was evaluated by measuring the absorbance (response) of the resulting mixtures using UV–visible absorption spectroscopy at 420 nm.

**Table 1:** Experimental independent variables at two levels used for the mycosynthesis of SNPs by the mycelial filtrate of selected isolate using Plackett Burman design

Variables	Symbol	Level	
		Low (− 1)	High (+ 1)
AgNO_3_ concentration	X_1_	1 Mm	2 mM
pH	X_2_	7	8
Incubation time	X_3_	3 days	5 days
Mycelial filtrate concentration	X_4_	10%	30%
Illumination	X_5_	Dark	Light

The following equation was used to determine each variable's main effect:

$${\text{Exi }} = \, {{\Sigma \,{\text{Mi}}^{ + } - \Sigma \,{\text{Mi}}^{ - } } \mathord{\left/ {\vphantom {{\Sigma \,{\text{Mi}}^{ + } - \Sigma \,{\text{Mi}}^{ - } } {\text{N}}}} \right. \kern-0pt} {\text{N}}}$$

where Exi was the variable’s main effect, Mi^+^ and Mi^**−**^ were the absorbance of the mycosynthesis of SNPs in trials where the independent variable (xi) was present at high and low levels, respectively, and N is the total number of trials divided by 2. A main effect figure with a^+^ ve sign means that the variable's maximum level is close to the optimum, whereas one with a negative sign implies that the variable’s minimal level is close to the optimum. Regression analysis and ANOVA were used to analyze the factorial experiment based on the Myco-SNPs. From the regression analysis, the significant variables were considered to have a greater effect on the Myco-SNPs [34]. For determining the significance of the variables, the statistical analysis (confidence level, t-value, and p-value) was computed using Microsoft Excel [35].

### Characterization studies for Myco-SNPs

#### Uv–Visible (Uv–Vis) spectroscopy

Using the mycelial filtrate of the selected isolate, the bio-reduction of AgNO_3_ occurred under optimal conditions of different factors for SNPs mycosynthesis. Visual observation of the color change from colorless to yellowish brown served as a preliminary confirmation of the production of SNPs. The absorption peak at wavelength 400–450 reveals the presence of silver nanoparticles [36].

#### Fourier transform infrared spectroscopy (FTIR)

The functional groups on the surface of the Myco-SNPs were examined using FTIR spectra. A Bruker alpha spectrophotometer (Perkin Elmer, USA) with a resolution of 4 cm^−1^ was used to conduct the FTIR analysis. A disc containing 50 mg KBr and 2% dried SNPs sample was prepared. The sample was scanned between 4000 and 400 cm^−1^, and the results were examined using the OPUS program.

#### X-ray diffraction spectroscopy (XRD)

Powder X-ray diffraction (XRD) pattern of nanoparticle powder was obtained with Cu-Kα1 radiation (1.5406 Å; 35 kV, 25 mA). The XRD pattern was analyzed to determine position, peak intensity, and width. The nanoparticle size was calculated using the Debye–Scherrer equation:

$${\text{D = }}{{{\text{k}}{.}\lambda } \mathord{\left/ {\vphantom {{{\text{k}}{.}\lambda } {\left( {\beta .\cos \theta } \right)}}} \right. \kern-0pt} {\left( {\beta .\cos \theta } \right)}}$$

where; D is the mean diameter of the nanoparticles, k is Scherrer constant (0.9), *λ* is the wavelength of monochromatic X-ray radiation source (*λ* = 1.5406 Å), *θ* is the Bragg diffraction angle and *β* is the full width of the peak at half maximum (FWHM) [37].

#### Transmission electron microscopy (TEM)

The size and morphology of Myco**-**SNPs were visualized using a JEOL JEM-2100 high-resolution transmission electron microscope at 200 kV. After drying a drop of aqueous SNPs on the carbon-coated copper, TEM grid samples were kept under vacuum in desiccators. By using imageJ software and Minitab statistical software (X64/21.3.1.0), the average size variation and diameter of nanoparticles were detected from TEM micrographs. The average and standard deviation were used to calculate values and size distributions.

### Antibacterial efficiency of Myco-SNPs

#### Minimum inhibitory concentration (MIC)

Four MDR clinical pathogens of burn wound infections from our previous studies were used to evaluate the antibacterial activities of Myco-SNPs using the resazurin microtiter dilution method using Muller Hinton broth (MHB) media following Clinical and Laboratory Standard Institutes [38]. These strains included *Pseudomonas aeruginosa* PA-09 [17], *Escherichia coli* EC-3 [18]*, Klebsiella pneumonia* KP-1 [19], and *Staphylococcus aureus* SA-17 [16]. Assays were performed three times in the 96-well microtiter plates. The wells were inoculated with each bacterial suspension (1 × 10^8^ CFU/mL), and the tested Myco-SNPs concentration ranges were 0.25–128 µg/mL. After incubation for 18 h at 37 ℃, each well received 20 µL of resazurin dye (0.1% w/v in distilled water), and the plates were kept for 1 h. The cells were considered actively metabolizing when the color of resazurin changed from blue or purple to pink. On the other hand, the appearance of blue indicated complete inhibition of bacterial growth. Two controls were used: (1) microorganisms inoculated into a 1 mM AgNO_3_ solution in MHB; and (2) culture medium (without inoculum) [39].

#### Time kill curve

Bacterial culture inocula were prepared in accordance with CLSI recommendations [38] for investigating the time kill study. The optical density of the organisms had been adjusted to 0.5 McFarland standard, and they had grown for 24 h in MHB media at 37 ℃. Another dilution was made to achieve an inoculum density of 5 × 10^5^ cells/mL. The flasks were then placed in an incubator for 90 min at 37 ℃ (EC-3, KP-1, SA-17) and for 120 min for PA-09 to grow the organisms to their respective log phases. Studies on the time kill curve were also carried out in accordance with CLSI recommendations. The optical density of each bacterium was adjusted after the initial lag phases, and test antibacterial agents were added at the MIC of the respective bacterium. Each flask was incubated at 37 ℃ at 150 rpm, and 0.1 mL aliquots were subcultured on Miller Hinton Agar (MHA) at every hour up to eight hours with suitable dilutions. The time kill curve was plotted as log_10_ CFU/mL vs. time (h).

#### Post agent efficiency (PAE)

Myco-SNPs (at 10 × MIC) were added to 1 mL of each bacterial suspension (10^7^ CFU/mL) in nutrient broth and then incubated at 150 rpm for 1 h at 37 ℃. The same set was repeated without using nanoparticles as a control. Cultures were then centrifuged after incubation for 5 min at 3000 rpm and then suspended again in 1 mL of MHB. One hundred microliters of each culture were added to 96-well plates and then incubated in a plate reader at 37 ℃ with an automated reading of absorbance every 10 min at 550 nm. PAE duration was determined in accordance with Stubbings et al. [40], i.e., the difference between the amount of time taken for antibacterial agent-treated cultures to reach 50% of the OD_max_ of the control culture and the needed time taken for the control culture to reach the same point. All steps were carried out three times.

### Assessment of the cytotoxic activity of Myco-SNPs

#### Cytotoxicity assay

The cytotoxicity of Myco-SNPs on the viability and morphology of the normal human skin fibroblast (HSF) cell line was examined using the SRB assay at Nawah Scientific Inc. (Mokatam, Cairo, Egypt), according to Bolla et al. [41]. On 96-well plates of microculture, 100 µL of HSF cells were incubated for 24 h. Then, another aliquot of 100 µL of media containing Myco-SNPs was applied to the cells, with concentrations ranging from 0.02 to 200 mg/mL. After 72 h of treatment exposure, cells were fixed by replacing media with 150 μL of 10% TCA and incubated at 4 ℃ for 1 h. When the TCA solution was withdrawn, distilled water was used to wash the cells five times. At room temperature for 10 min, 70 μL of SRB solution (0.4% w/v) were added and incubated in the dark. Then, 1% acetic acid was used to wash the plates three times and leave them to dry in the air. After that, 150 mL of Tris (10 mM) was added to break up the protein-bound SRB stain. Microscopic examination was done, and observations were noted. Absorbance was measured using a BMGLABTECH^®^-FLUO star Omega microplate reader (Ortenberg, Germany) at 540 nm. The cells were treated with DMEM alone as a negative control, while 1% DMSO and 10% DMSO were used as vehicle and positive controls, respectively. The percentage growth inhibition was calculated using the following formula, and the concentration of Myco-SNPs needed to inhibit cell growth by 50% (IC_50_) was generated from the dose–response curves.

$${\text{Inhibition }}\% = 100 - \left\{ {{{\text{mean of individual tested group}} \mathord{\left/ {\vphantom {{\text{mean of individual tested group}} {\text{mean of control group}}}} \right. \kern-0pt} {\text{mean of control group}}}} \right\} \times 100$$

### Statistical analysis

All experimental data was replicated three times, and the average values were expressed ± SD. One-way analysis of variance (ANOVA) by GraphPad Prism v. 5.01 and Microsoft Excel 2010 for experimental designs and statistical analysis to illustrate the relation between response and effect of variables were used in this study.

## Results and discussion

Mycosynthesis of SNPs has many advantages due to the easy achievement of optimal conditions, being inexpensive, non-toxic, and scaling up [42, 43], in contrast to chemical and physical synthesis. Due to the unique physio-chemical characteristics of SNPs, they have been incorporated into different industrial and medicinal applications [44]. The first step in the present study was to screen for the most potent Myco-SNPs producers from four endophytic fungi isolated from the leaves of *L. shawii*.

### Screening for extracellular production of Myco-SNPs

**Fig. 1:**
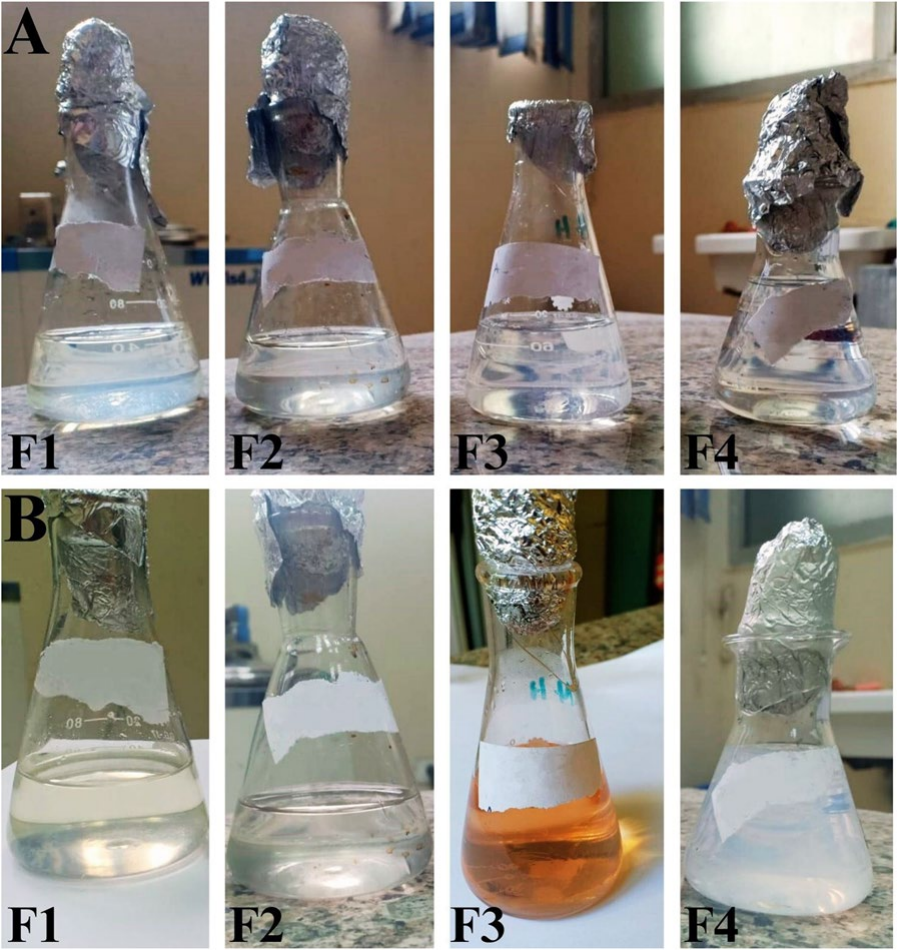
Visual observation of the mycosynthesis of SNPs by different mycelial filtrates of tested endophytic fungi. At 0 time after addition of AgNO_3_ 1 mM solution (**A**), Color change reaction after exposure to AgNO_3_ solution for 24 h (**B**)

**Fig. 2:**
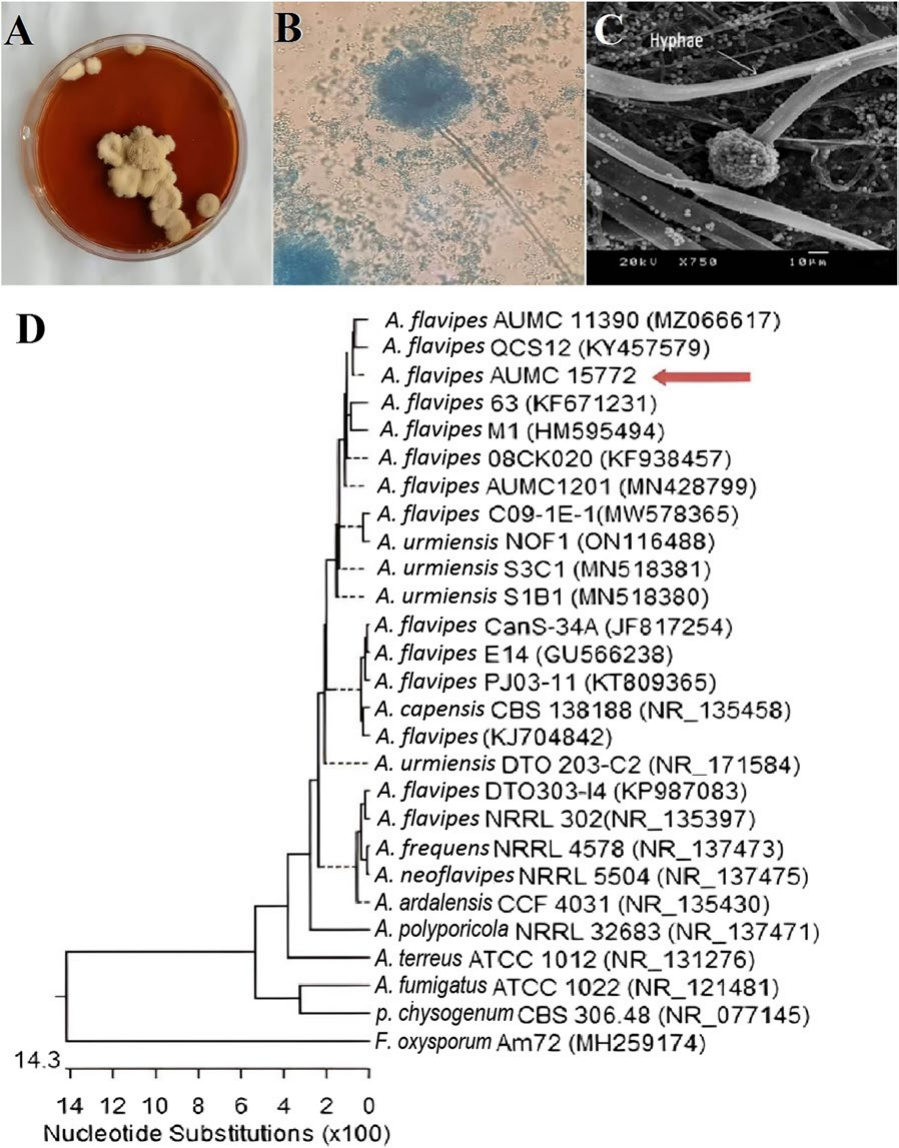
Morphological and molecular identification of *A. flavipes* AUMC 15772. Fungal colonies on SDA after 5–7 days at 30 ℃ (**A**). Conidial head and conidiophores at × 40 and × 750 magnification by LM (**B**) and SEM, respectively (**C**). Phylogenetic relationship between *A. flavipes* AUMC 15772 and other fungal strains retrieved from database showed 100–93% identity with several strains of *A. flavipes* based on partial 18S rRNA gene sequences from Genebank, and our strain is mentioned against red arrow (**D**)

A common technique for testing microbiological isolates for the production of silver nanoparticles is color change observation [45]. The extracellular mycosynthesis of SNPs was evaluated by using the mycelial filtrate of each fungus and observing color changes in the presence of 1 mM AgNO_3_ [35]. The screening revealed that the most potent Myco-SNPs producer was F3, in which the color of samples converted from colorless to yellowish brown after 24 h of mycelial filtrate incubation with AgNO_3_ (Fig. 1). The observed results were similar to those of Abd Elnaby et al. [46], who claimed that the actinomycetes’ production of SNPs was indicated by a shift in color to yellowish brown. Similar findings have been made by Abdelmoneim et al. [14], who recorded that the synthesis of SNPs led to a shift in color to brown after incubating *Leclercia adecarboxylata* supernatant cultures with AgNO_3_. Next, as part of the initial validation, this finding was additionally examined spectrophotometrically at 300–500 nm [35], giving a characterized absorption peak at 420 nm (Fig. 2), which confirms the formation of SNPs. It has been demonstrated that UV–Vis spectroscopy-based technique is a useful tool for confirming the production of nanoparticles [47]. Our results revealed that the selected isolate F3 was the most potent Myco-SNPs producer and was used throughout the rest of the work.

### Identification of the most potent Myco-SNPs producer (F3)

#### Morphotypic identification

**Fig. 3:**
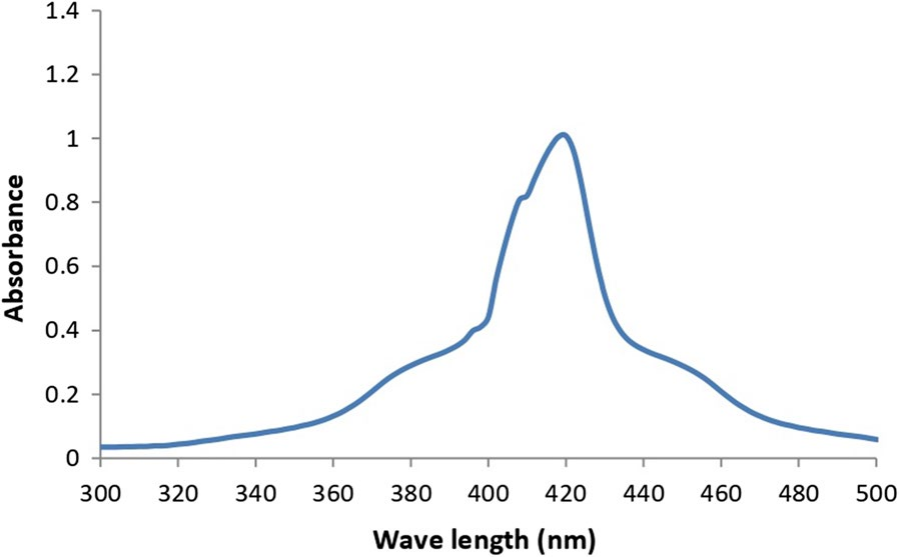
UV–visible spectra of Myco- SNPs by F3 isolate. The absorption of Myco-SNPs was recorded after 24 h of incubation and exhibited a strong peak at 420 nm

The most potent Myco-SNPs producer (F3) appeared macroscopically as irregularly furrowed buff-yellowish colonies with a red-brown reverse on SDA medium (Fig. 3A). After light and scanning microscopic examinations, the conidial heads of F3 appeared loosely columnar to radiate with sub-globose vesicles (Fig. 3B, C).

#### Molecular identification

The most potent endophytic fungus F3 that resulted in the production of SNPs was molecularly identified as *A. flavipes* AUMC 15772 and deposited in GenBank with Accession No. OP521771. The phylogenetic tree of selected fungi is shown in Fig. 3D. This technique is considered the most accurate method for the identification of microorganisms [48]. As far as is known, there are many biological activities of secondary metabolites in endophytic fungi [15]. However, no research has been conducted on the mycosynthesis of SNPs from endophytic *A. flavipes* AUMC 15772. Therefore, the present study is the first to investigate the biological activities of Myco-SNPs from endophytic *A. flavipes* AUMC 15772 to be used in further medical and pharmaceutical applications.

#### Optimization for SNPs mycosynthesis

To the best of our knowledge, the optimization of Myco-SNPs from endophytic *A. flavipes* AUMC 15772 for large-scale production has not been reported until now. Therefore, OVAT and Plackett–Burman experimental designs were conducted to detect the most optimal variables influencing Myco-SNPs production.

#### One variable at time (OVAT) method

Optimization of physiochemical properties will increase product yield in addition to supporting a higher rate of growth. The growth parameters, such as temperature, pH, AgNO_3_ concentration, and mycelial filtrate concentration, play an important role in controlling enzyme efficiency, which affects SNPs mycosynthesis [49].

#### Effect of AgNO_3_ concentrations

**Fig. 4:**
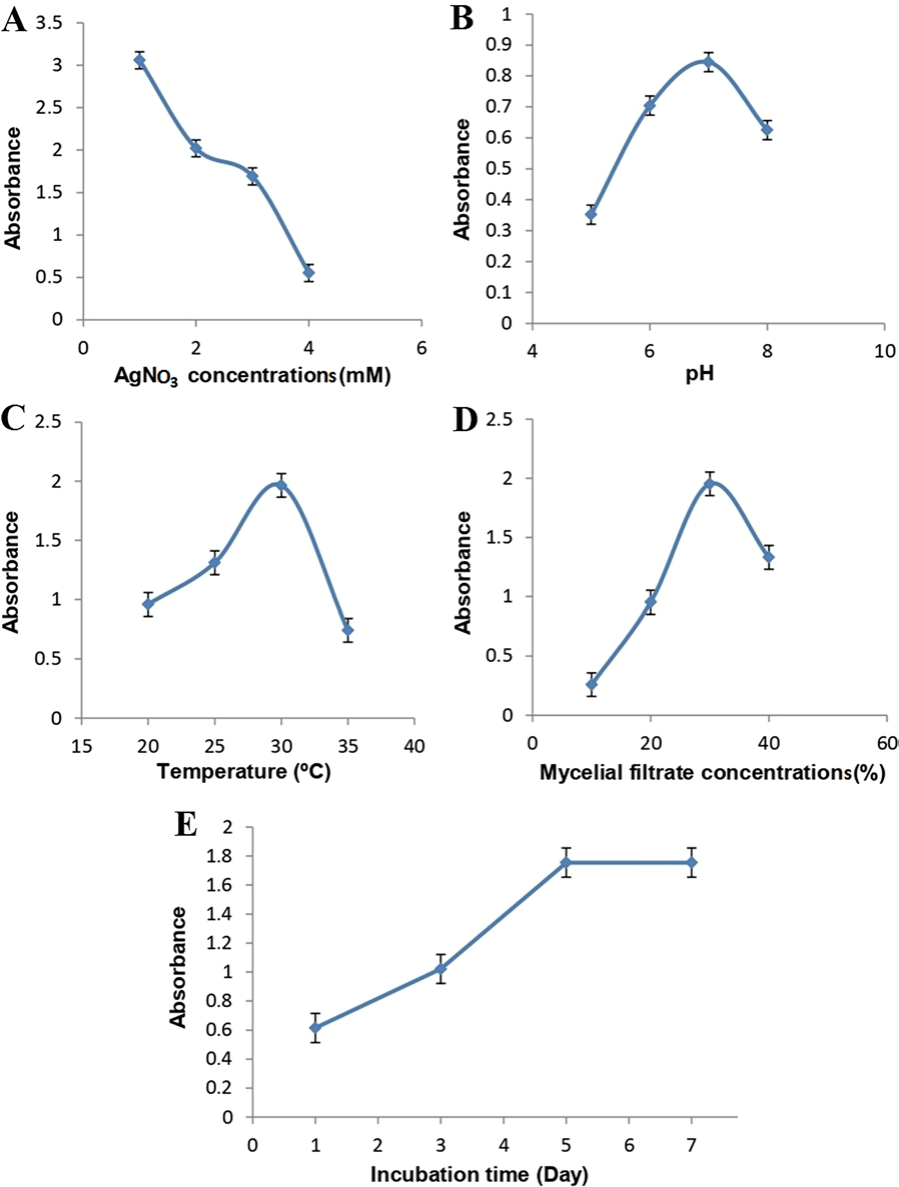
OVAT optimization parameters on the mycosynthesis of SNPs from *A. flavipes* AUMC 15772. Effect of AgNO_3_ concentrations (**A**). pH values (**B**). Temperature (**C**). Mycelial filtrate concentrations (**D**). Incubation time (**E**)

Mycosynthesis of SNPs with varying concentrations of AgNO_3_ solution from 1 to 4 mM from *A. flavipes* AUMC 15772 was investigated, and the absorbance of the produced SNPs solutions was recorded spectrophotometrically. The optimal concentration of AgNO_3_ for current OVAT optimization was investigated at 1 mM. This was reflected by a color shift and an increase in absorption at 420 nm (Fig. 4A). When the AgNO_3_ concentration is increased to 4 mM, this may lead to an increase in particle size and aggregation of Myco-SNPs [50]. Therefore, the most stable synthesis of Myco-SNPs was discovered at 1 mM of AgNO_3_ without aggregation for longer [51]. Our findings were similar to those of Banu et al. [52], who utilized 1 mM AgNO_3_ for SNPs biosynthesis by using a fungal extract of *Rhizopus stolonifer*. In contrast to Abdelmoneim et al., who recorded that the highest biosynthesis of SNPs was noticed by increasing AgNO_3_ concentration to 6.0 mM [14], This variation may be due to the differences between microorganisms, as the optimum AgNO_3_ depends on fungal filtrate composition, which differs from strain to another [53]. Moreover, the optimal concentration in many cases of fungal biosynthesis of SNPs is 1 mM AgNO_3_ [54].

#### Effect of pH

By using spectrophotometric analysis, the impact of changing pH levels on the optimum and stable synthesis of Myco-SNPs was observed. It has been shown that variations in pH have an impact on the size and structure of nanoparticles because pH can modify the charge of molecules, which may impact both their stabilizing and capping properties [55, 56]. Our findings indicated that pH 7 was the optimal point for Myco-SNPs formation. According to these findings, neutral medium, as shown in (Fig. 4B), was preferable to acidic or alkaline media for the mycosynthesis of SNPs. The protein structure was impacted at minimal pH levels [36], in which the protein lost its function and became denatured, which caused the nanoparticles to aggregate [57].Our results were in agreement with Sarsar et al. [29], who discovered that the highest peak at pH 7 was noticed for the production of SNPs. Also, our findings agreed with Banu et al. [57], who found that maximum absorbance occurred at pH 7. Moreover, Jain et al. [58] used pH 7 for the production of SNPs from *A. flavus.*

#### Effect of temperature

The nucleation mechanism that occurs during the production of Myco-SNPs is significantly influenced by temperature, as temperature is one of the most important factors affecting the rate of reaction [27]. The results showed that the rate of SNPs mycosynthesis increased as the temperature of the reaction mixture rise to reach maximum at 30 ℃ (Fig. 4C) and then declined at higher temperatures due to the decrease in enzyme activity, which affects the rate of mycosynthesis. Our results were in agreement with Mittal et al. [59], who recorded that SNPs biosynthesis was increased when the temperature was 25 ℃. Our results revealed that temperatures below and above 30 ℃ decrease the rate of mycosynthesis of SNPs. In contrast, another study conducted at a different optimum temperature of 60 ℃ using *Penicillium oxalicum* [60]. This may be due to the inactivation of biomolecules that have an important effect on the biosynthetic pathway.

#### Effect of mycelial filtrate concentration

The majority of fungal mycelial filtrates are capable of mycosynthesis of SNPs as they act as capping and reducing agents for the formation of nanoparticles [61, 62]. As seen in Fig. 4D, Myco-SNPs production increased when the mycelial filtrate concentration of *A. flavipes* AUMC 15772 was increased from 10 to 30%. As a result, the concentration of the mycelial filtrate at 30% was selected as the optimal concentration for SNPs mycosynthesis, which decreased at higher concentrations as higher amounts of reducing agents caused the synthesis of large molecules and thus the aggregation of the Myco-SNPs [63]. Our findings were in accordance with Aboelfetoh et al. [64], who recorded that 20% biomass of *Caulerpa serrulata* extract promoted the better synthesis of SNPs, and an additional increase in the extract percentage decreased the synthesizing efficiency.

#### Effect of incubation time

Incubation time is an important parameter to steer the conditions of reaction and adjust the shape and size of nanostructures [65]. Figure 4E showed the time dependence of Myco-SNPs using a 1 mM AgNO_3_ solution. It revealed that as reaction time increased, the color of the resulting solutions changed and absorption increased, indicating the formation of more Myco-SNPs up until day 5, which demonstrates the stabilization of Myco-SNPs solution [66]. Day 5 was considered the optimum time for Myco-SNPs production from* A. flavipes* AUMC 15772. Similarly, Elamawi et al. [67] recorded that the maximum SNPs production similarly at 4 to 5 days of incubation time. On the other hand, Abdelmoneim et al. [14] recorded that the highest production of bio-SNPs was accomplished during 2 days of incubation duration. Several researchers have noted different SNPs biosynthesis incubation times, such as 3 days [68].

### Plackett burman design (PBD) for optimization of Myco-SNPs

**Fig. 5:**
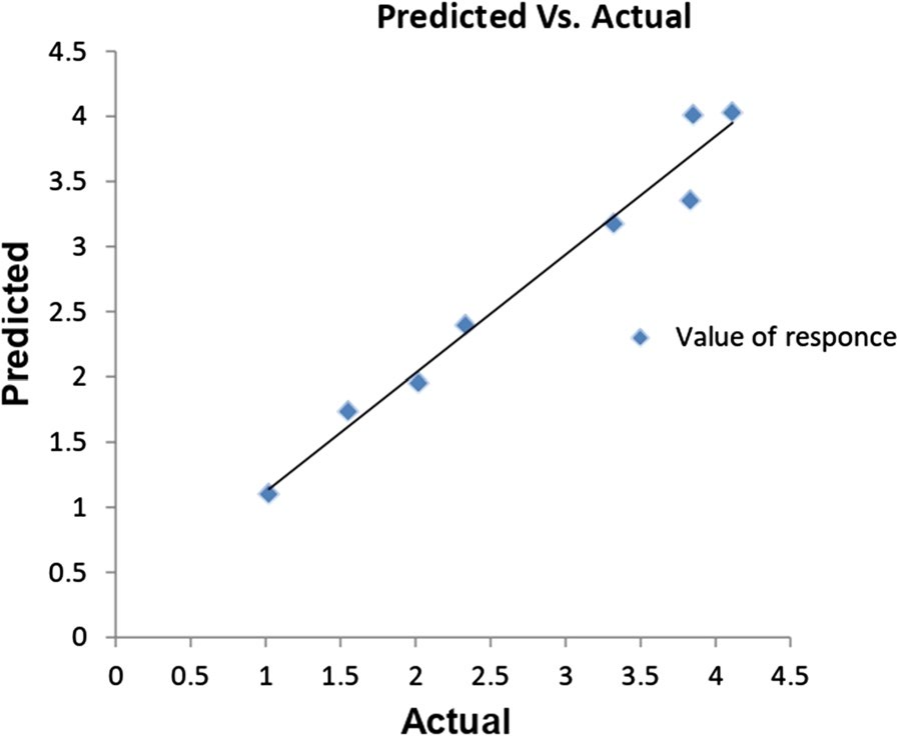
The parity plot of the correlation between actual/experimental and predicted responses of the Myco-SNPs by mycelial filtrate of *A. flavipes* AUMC 15772

**Fig. 6:**
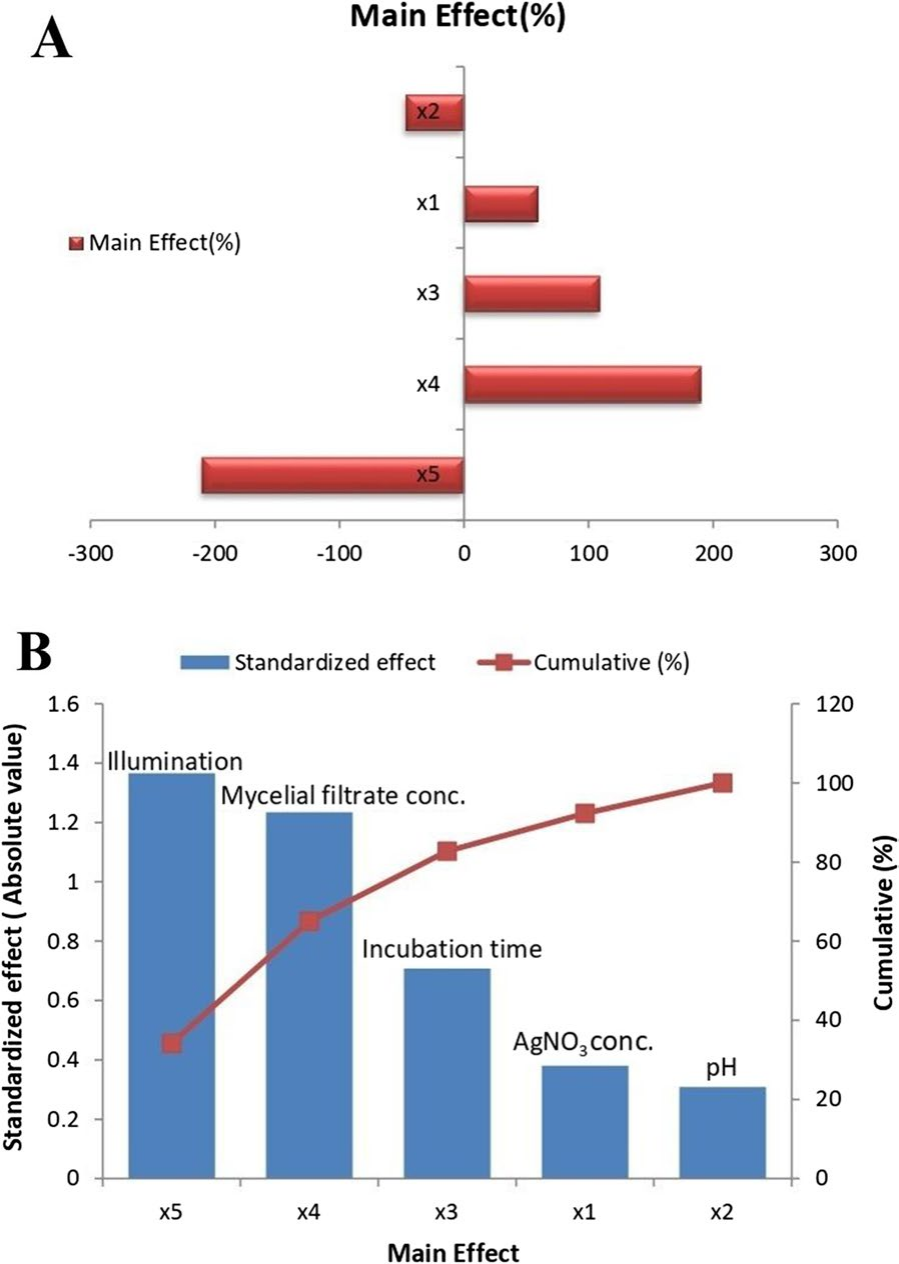
Effect of independent variables according to Plackett Burman experimental results on the mycosynthesis of SNPs from *A. flavipes* AUMC 15772. The main effects of the process variables where x_1_: AgNO_3_ concentration, x_2_: pH, x_3_: incubation time, x_4_: mycelial filtrate concentration, x_5_: illumination (**A**). Pareto chart showing significance of each variable affecting the mycosynthesis of SNPs (**B**)

Plackett–Burman design, compared to other statistical techniques, is much simpler and takes less time [69]. This design was employed to determine the most optimal levels of variables regulating the mycosynthesis of SNPs from the mycelial filtrate of *A. flavipes* AUMC 15772 [35]. The absorbance (response) of the resultant solutions was examined spectrophotometrically at 420 nm in order to ascertain the mycosynthesis of SNPs. Table 2 showed the actual and predicted response for the production of Myco-SNPs for eight different trials involving combinations between five independent variables at the absorbance range of 1.122–4.049 at 420 nm, as shown in the parity plot (Fig. 5). The relationship between the response and different independent factors was detected by a multiple regression mathematical model using Microsoft Excel 2010 for evaluating confidence level*,* p-value, and* t*-value. Table 3 displayed the analysis of t-values and the regression coefficients for five variables. The coefficients of each variable showed the degree to which it influences the mycosynthesis of SNPs in a positive or negative way. The mycosynthesis of SNPs is stronger at a high level of the investigated factor when the effect's sign is positive. SNPs mycosynthesis is stronger at a low level of the factor when the sign is negative. To establish the statistical significance of the response and the main effects on SNPs production, the t-values for each factor were calculated. The greater the magnitude (*t*-values = 33.5436), the greater the evidence of a significant difference. The closer the t-values are to 0, the less significant the difference. According to R^2^ coefficient of determination of 0.9874, the independent factors were responsible for 98.74% of the sample variance in the production of Myco-SNPs, and only 1.26% of the total variations were not explained by independent factors. The coefficient of detection (adjusted R^2^) was calculated to be 0.9560, which is very high, which indicates high significance of the model and demonstrates a well-matched match of actual values with the predicted values of Myco-SNPs production. Among 5 variables, AgNO_3_ concentration, mycelial filtrate concentration, and incubation time showed a positive sign of the effect on the mycosynthesis of SNPs. while pH and illumination showed negative signs of the effect (Fig. 6A). In Fig. 6B, the standardized Pareto chart of the main effects indicated the order of the effects on SNPs production. The Pareto chart indicated that AgNO_3_ concentration, mycelial filtrate concentration, and incubation time were the most important significant variables affecting on SNPs mycosynthesis, while pH was the most insignificant variable. Table 3 illustrated that the model was significant with confidence intervals greater than 95%, according to the significance values of p < 0.05 (0.0311). This means that higher levels of AgNO_3_, mycelial filtrate concentration, and incubation time with low pH and in dark conditions can promote the mycosynthesis of SNPs from mycelial filtrate of* A. flavipes* AUMC 15772.

**Table 2:** Plackett Burman design matrix with observed and predicted values of five independent variables and the absorbance readings at 420 nm as a response reflecting the Myco-SNPs concentration

Trials	X_1_	X_2_	X_3_	X_4_	X_5_	Responses	
						Actual*	Predicated
1	1	− 1	− 1	1	− 1	3.848	4.0085
2	1	1	− 1	− 1	1	1.122	1.09975
3	1	1	1	− 1	− 1	3.151	3.17325
4	− 1	1	1	1	− 1	4.049	4.02675
5	1	− 1	1	1	1	3.511	3.3505
6	− 1	1	− 1	1	1	1.931	1.95325
7	− 1	− 1	1	− 1	1	1.575	1.7355
8	− 1	− 1	− 1	− 1	− 1	2.554	2.3935

Where the five variables (X_1_-X_5_) are in order, AgNO_3_ concentration, pH, incubation time, mycelial filtrate concentration and illumination, respectively. For each variable, − 1 represents the low concentration level and + 1 represents the high concentration level

^*^The experimental values were the mean absorption replicates at 420 nm

**Table 3:** Regression statistical analyses of the Plackett–Burman experimental results

Variable	Coefficient	Standard error	Main effect	Main effect (%)	tStat	P-value	Lower 95.0%	Upper 95.0%
Intercept	2.7176	0.0810	–	–	33.5436	0.0008	2.3690	3.0662
x_1_	0.1903	0.0810	− 1.36575	− 210.6826	2.3498	0.1432	− 0.1582	0.5389
x_2_	− 0.1543	0.0810	1.23425	190.3972	− 1.9054	0.1970	− 0.5029	0.1942
x_3_	0.3538	0.0810	0.70775	109.1785	4.3678	0.0486	0.0052	0.7024
x_4_	0.6171	0.0810	0.38075	58.7350	7.6171	0.0168	0.2685	0.9657
x_5_	− 0.6828	0.0810	− 0.30875	− 47.6282	− 8.4287	0.0137	− 1.0314	− 0.3342

Analysis of variance (ANOVA)						
	df	SS	MS	F-test	Significance F	P-value
Regression	5	8.2597	1.6519	31.4591	0.0311	significant
Residual	2	0.1050	0.0525			
Total	7	8.3647				
R square:	0.9874					
Adjusted R square:	0.9560					
Multiple R:	0.9937					

Where *t* Student’s test, *P* corresponding level of significance, df degree of freedom, *SS* sum of squares, *MS* mean sum of squares, *F* Fisher’s function; and significance. *F* corresponding level of significance

After PBD optimization, the overall optimal conditions for increasing mycosynthesis of SNPs from mycelial filtrate of *A. flavipes* AUMC 15772 were 2 mM AgNO_3_ concentration, 30% mycelial filtrate concentration, 5 days of incubation, and pH 7 in dark conditions. In a related investigation by Abdelmoneim et al. [14], five variables were examined by PBD to establish the impact of the factors on SNPs biosynthesis by using the cell supernatant of *Leclercia adecarboxylata*. They reported that pH, AgNO_3_ concentration, and illumination were the most significant variables affecting on SNPs biosynthesis from bacteria, and the time of incubation was the most insignificant variable. Also, Trivedi et al. [70], who used citrus peel extract to examine the impact of some factors on SNPs biosynthesis, recorded that temperature was the most significant variable impacting SNPs biosynthesis, followed by illumination and pH. While, AgNO_3_ concentration was an insignificant variable.

### Characterization for Myco-SNPs

#### UV–visible spectroscopy

**Fig. 7:**
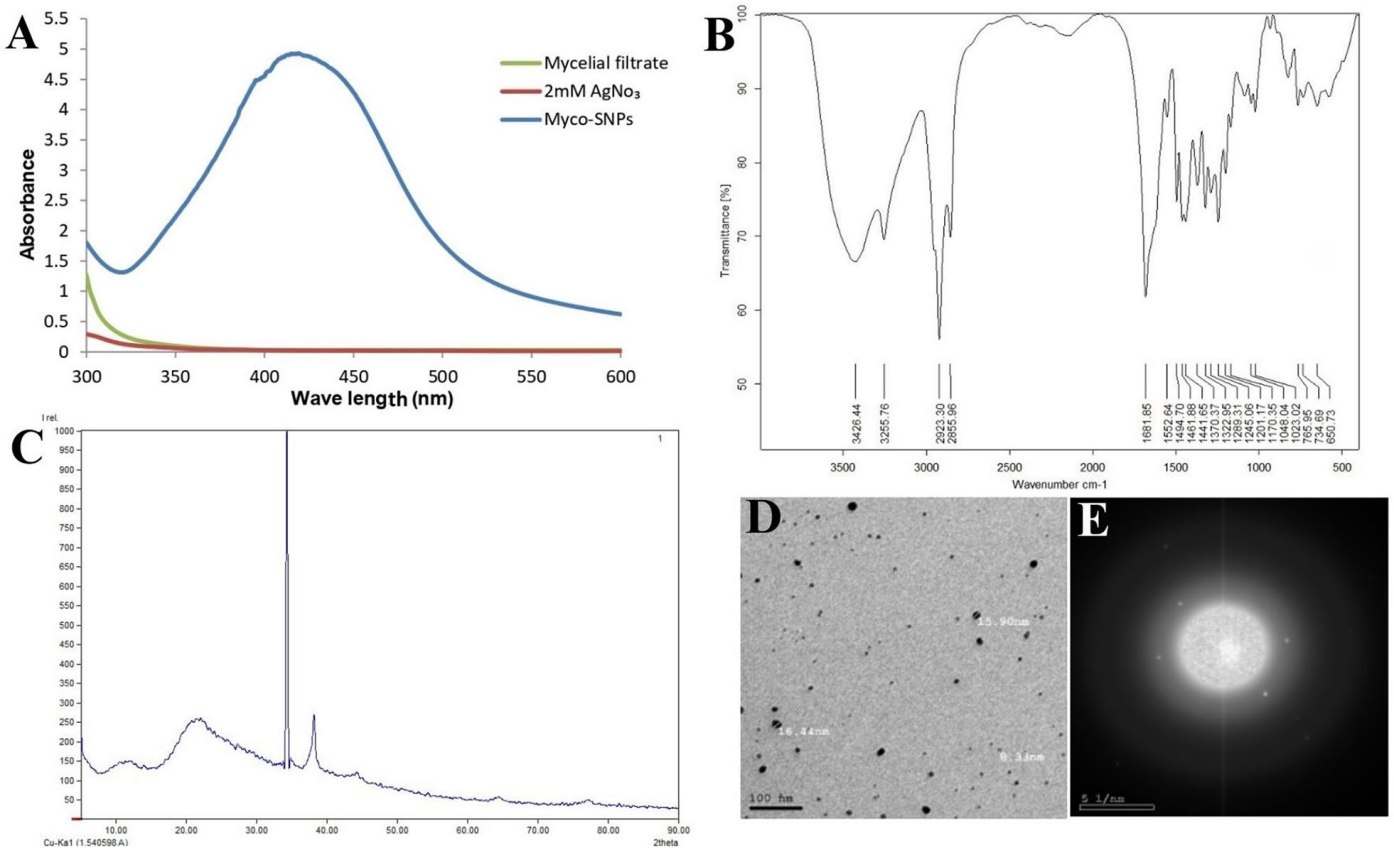
Physiochemical characterization of Myco-SNPs from *A. flavipes* AUMC 15772. UV–vis absorption spectra of Myco-SNPs, AgNO_3_, and the mycelial filtrate of *A. flavipes* AUMC 15772 (**A**). FTIR spectrum of Myco-SNPs (**B**). X-ray diffraction pattern of Myco-SNPs (**C**). TEM image at magnification 100 nm (**D**) and Selected-Area Electron Diffraction (SAED) patterns of Myco-SNPs (**E**)

To confirm the mycosynthesis of SNPs, the sample of *A. flavipes* AUMC 15772 underwent UV–Vis spectroscopy examination. Figure 7A showed a single and strong peak at 420 nm for the Myco-SNPs under optimal conditions compared to AgNO_3_ and mycelial filtrate of *A. flavipes* AUMC 15772. Similar findings were made by Ninganagouda et al. [71], who found the absorption peak of SNPs between 380 and 450 nm. Also, Sunkar and Nachiyar [72] detected Myco-SNPs from endophytic fungi isolated from leaves of *Aravae lanata* and* Garcinia Xanthochymus* and had absorption peaks at 423 nm and 400 nm.

#### Fourier transform infrared spectroscopy (FT-IR)

The FTIR spectrum of the SNPs indicated the different functional groups that participated in stabilization and biosynthesis of the nanoparticles, and this has a highly important role in characterizing the proteins binding with the SNPs and can detect the secondary structure of metal nanoparticles with protein interaction [58]. The interaction of biological components of *A. flavipes* AUMC 15772 mycelial filtrate with nanoparticles indicated peaks at 3426.44, 2923.30, 1681.85, and 1552.64 cm^−1^ (Fig. 7B). The broad spectrum at 3426.44 showed the presence of polyphenols, and the absorption peak that was detected at 2923.30 cm^−1^ suggested the binding of silver ions with the OH group. Also, the presence of C = C stretch at 1681.85 cm^−1^ indicated the presence of a broad range of alkene groups in the mycosynthesized nanoparticles. Moreover, the band at 1552.64 cm^−1^ could be possible due to N–O asymmetric stretching, illustrating the active involvement of nitro compounds. Other peak at 1023.02 cm^−1^ illustrated the presence of aliphatic amines because vibrations of the C-N functional group, which are generally found in proteins, are involved in the reduction of the metal ions. The presence of distinctive functional groups such as phenols, aldehydes, nitro compounds, and alcohols as bioconstituents was present in mycelial filtrates of *A. flavipes*, which contribute to the bioreduction process for SNPs biosynthesis. Also, earlier publications provide substantial support for the current findings [73, 74].

#### X-ray diffraction (XRD) spectroscopy

The nanoparticles synthesized in this method were characterized using powder XRD to confirm the particles as silver and to know the structural information. Figure 7C showed XRD pattern of the Myco-SNPs. The XRD pattern of SNPs did not detect any impurity peaks and revealed a pure single phase (JCPDS card No. 00-004-0783). The XRD pattern revealed strong XRD reflections at 2θ = 34.23° and 38.18°, suggesting the formation of SNPs with good crystal quality. By applying Scherer’s equation, the average crystal size (36.47 nm) of Myco-SNPs was calculated. These results were nearly identical to those of Rajesh et al. [75], who calculated the average crystal size of synthesized silver nanoparticles by *Lactobacillus acidophilus* culture filtrate (33 nm) by applying Scherer’s equation.

#### Transmission electron microscopy (TEM)

**Fig. 8:**
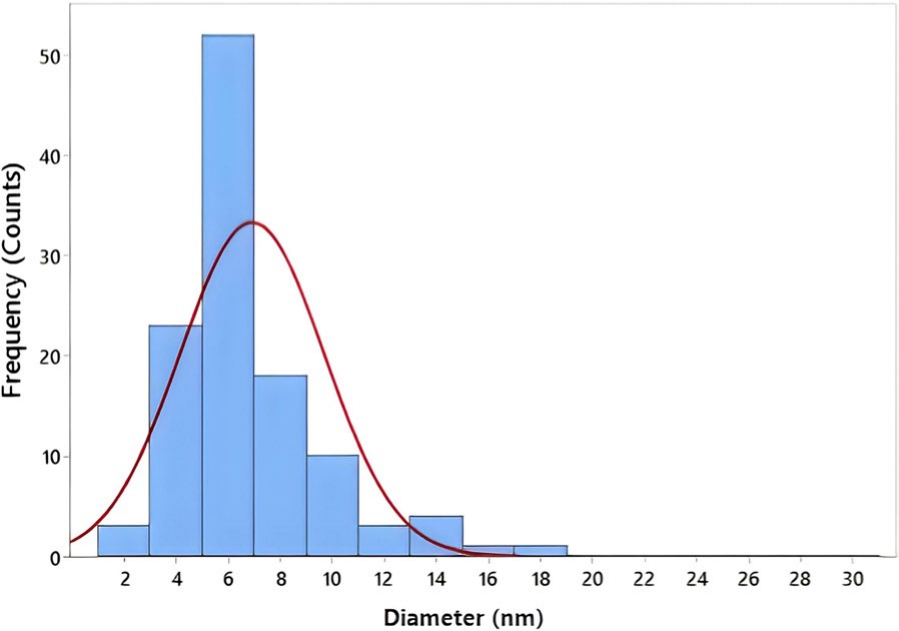
Histogram of the size distribution of Myco-SNPs

TEM is used to examine the distribution, shape, and size of the nanoparticles [76]. Analysis of Myco-SNPs from *A. flavipes* AUMC 15772 by TEM proved the size of SNPs to be nano-scale, spherical-shaped, with smooth edges, and separated without any aggregation (Fig. 7D). Selected area electron diffraction (SAED) patterns exhibited concentric rings with intermittent dots, indicating that these nanoparticles are crystalline in nature (Fig. 7E), with similarity to that reported by Murthy et al. [77]. The results indicated that Myco-SNPs have well-dispersed SNPs. These results were similar to those of Elamawi [67], who indicated that the size of SNPs ranged from 1 to 25 nm. The same type of nanoparticles with variable shapes and sizes was observed in common biological systems in the range of 3–30 nm when synthesized by *A.niger* [78]. In agreement with our findings, Lotfy et al. [35] recorded that the mycosynthesized SNPs observed by using mycelial filtrate of *A. terreus* had a spherical shape with dimensions between 7 and 23 nm. By using ImageJ software, our data detected that Myco-SNPs particle sizes ranged from 2.842 to 17.700 nm, with an average of 6.9232 nm and a standard deviation of 2.7628, as shown in the histogram (Fig. 8).

#### Antibacterial efficiency of Myco-SNPs

Mycoynthesised AgNPs were studied for their antibacterial efficiency against four selected MDR bacterial strains: three Gram-negative bacteria: *Pseudomonas aeruginosa* PA-09, *Escherichia coli* EC-3, and* Klebsiella pneumonia* KP-1, and one Gram-positive: *Staphylococcus aureus* SA-17. Myco-SNPs showed high and significant efficiency against selected MDR wound pathogens, which were slightly varied. The most affected pathogen was PA-09, followed by KP-1, EC-3, and SA-17, respectively.

#### Minimum inhibitory concentration (MIC)

The antimicrobial effect of Myco-SNPs was illustrated in Table 4 by determining MIC values. We noticed that Myco-SNPs have MICs in the range of 8–32 µg/mL. PA-09 and KP-1 were observed to be the most sensitive strains, having the lowest MIC (8 µg/mL).While SA-17 was observed to be the least sensitive strain, recording the highest MIC (32 µg/mL). In contrast to earlier studies, the MIC of* E. coli*, *P. aeruginosa,* and *B. subtilis* was 84.28, 74.26, and 94.43 µg/mL, respectively [79]. Likewise, the range of MICs for *K. pneumonia* [75] and *S. aureus* [14] was 60 and 500 µg/mL, respectively. These differences in MIC values are attributed to the colloidal state, concentration, and size of Myco-SNPs [80]. The small sizes of nanoparticles are more toxic to pathogens than the large sizes due to their easy diffusion, and they were most effective at sizes smaller than 50 nm [81]. Gram-negative strains were effectively inhibited by Myco-SNPs, which is in agreement with earlier reports [82, 83]. This has been described due to the structural difference in the bacterial cell walls between Gram-negative and Gram-positive bacteria, which have a thick layer of peptidoglycan [84, 85]. However, several findings contradicted this conclusion [86, 87] or recorded varying sensitivity with these bacterial strains [88, 89].

**Table 4:** Minimum inhibitory concentration (MIC) and post agent efficiency (PAE) of Myco-SNPs

Bacteria	MIC of Myco-SNPs (µg/mL)	PAE of Myco-SNPs (in hours at 10 × MIC)
PA-09	8	9 ± 0.6
KP-1	8	5 ± 0.3
EC-3	16	5 ± 0.5
SA-17	32	8 ± 0.6

The data were expressed as mean ± standard deviation (n = 3).*p ≤ 0.05 when compared with control

#### Time kill curve

**Fig. 9:**
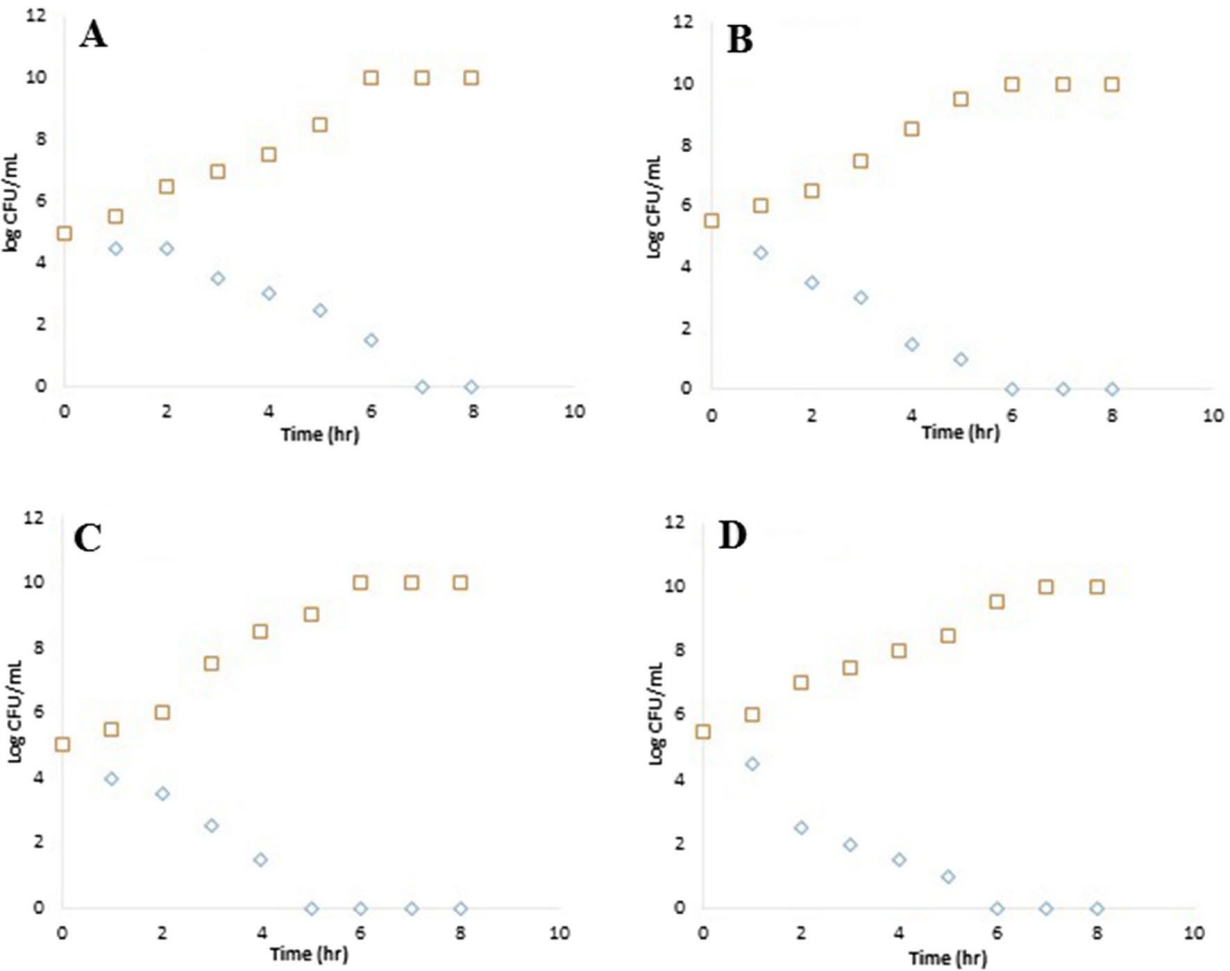
Time kill curve of four MDR bacteria: *P. aeruginosa* (PA-09) (**A**); *K. pneumonia* (KP-1) (**B**); *E. coli* (EC-3) (**C**) and *S. aureus* (SA-17) (**D**) incubated with Myco-SNPs (square) compared to control without SNPs (diamond). The values represented the mean ± SD of three individual observations. *p ≤ 0.05 when compared with control

After incubating Myco-SNPs with 10^5^ CFU/mL of tested bacterial organisms, there was a decrease in bacterial growth rate as compared to the control. Also, all tested strains showed complete inhibition after 5–6 h (Fig. 9). The mode of action of Myco-SNPs as antibacterial may be due to the penetration of nanoparticles into the bacterial cell wall, affecting their ability to anchor, which is mainly responsible for the structural alterations of the membrane and finally leads to cell death [90]. In addition, it was reported that Ag^+^ ions inactivate the cell respiratory enzymes of *E. coli* [91], as numerous bacterial enzymes are inactivated due to the firm interaction of Ag^+^ ions released from silver nanoparticles with SH^−^ groups, which is the major structural part of the enzyme conformation [92, 93]. Moreover, SNPs cause inhibition of bacterial DNA in *S. aureus* and* P. aeruginosa* [94]. This could be due to the reaction of SNPs with the phosphorus and sulfur groups, which interfere with DNA replication, causing the microbial system to collapse [95, 96].

#### Post agent efficiency (PAE)

The study of post-agent efficiency (PAE) was performed to detect the time of inhibited growth after exposure to nanoparticles. PAE was extremely beneficial in estimating the antibacterial efficiency of nanoparticle materials and drugs [97, 98]. Thus, our study used 10 times the MIC for PAE detection. All tested organisms have shown post-agent effects, with the highest PAE observed in the case of PA-09, followed by SA-17 (Table 4). The growth of PA-09 remained inhibited after their exposure to nanoparticles for a longer time (9 h) and SA-17 (8 h). While both KP-1 and EC-3 inhibited only for 5 h. It was shown that Myco-SNPs exhibited high PAE in all selected pathogens, illustrating that Myco-SNPs are harmful to these pathogens, preventing them from remaining viable for a longer period of time.

#### Cell viability and cytotoxic activity

**Fig. 10:**
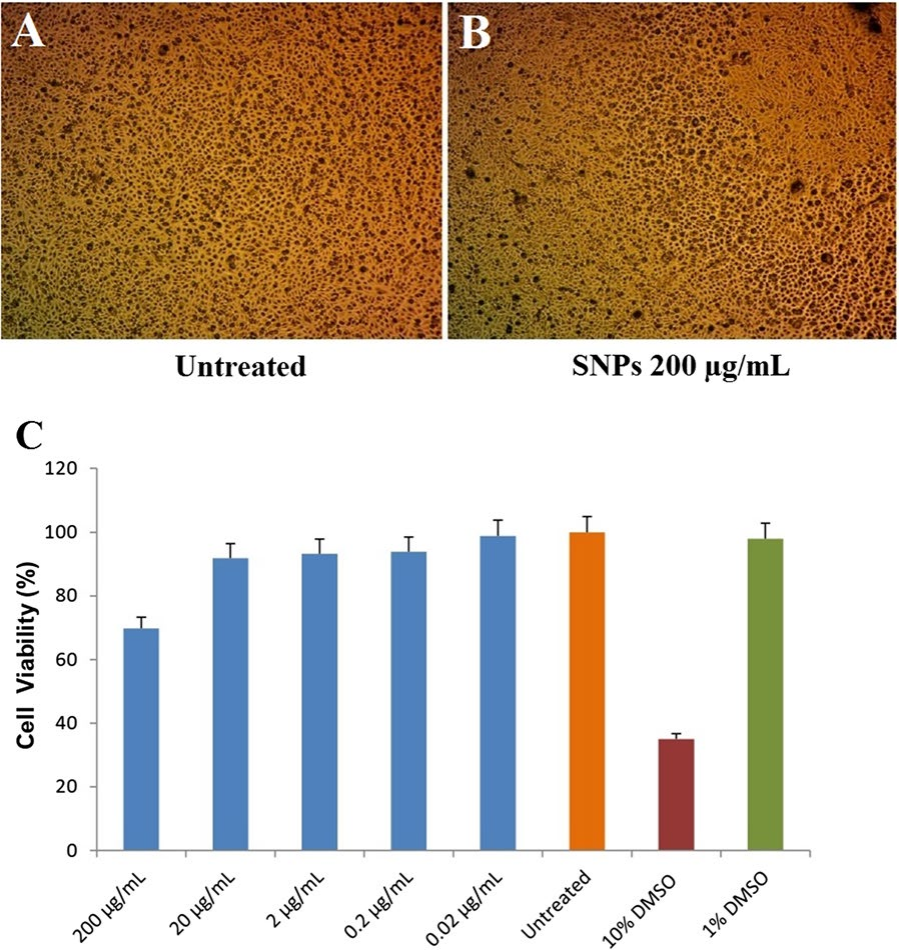
Cytotoxicity evaluation of Myco-SNPs. Representative images with magnification of (× 10) taken by light inverted microscopy for untreated HSF cells (**A**) and treated with 200 μg/mL of Myco-SNPs at 48 h (**B**). Skin fibroblast cells were treated with different concentration of Myco-SNPs and cell viability was examined by SRB assay (**C**). Experiment was repeated triplicate and the data were statistically analyzed with GraphPad Prism software

Based on the high antimicrobial activity of Myco-SNPs against MDR wound pathogens, it is important to confirm the cytotoxicity of Myco-SNPs as a novel fungal-based drug that may be used in wound healing, as one of the most important characteristics of biomaterials that are used in wound healing is cell viability [99]. Therefore, the cytotoxicity of Myco-SNPs was tested on HSF cells. The results revealed that Myco-SNPs were highly biocompatible (not toxic) with IC_50_ < 200 µg/mL (Fig. 10).

## Conclusion

In this study, a novel endophytic fungus (*A. flavipes* AUMC 15772) isolated from leaves of *L. shawii* showed its ability to produce Myco-SNPs with a characteristic peak at 420 nm. The physiochemical properties of Myco-SNPs were characterized by UV–visible spectroscopy, FT-IR, XRD, and TEM. PBD was used to optimize the mycosynthesis of SNPs and showed that AgNO_3_ concentration, mycelial filtrate, and incubation time have a significant positive effect on the production of Myco-SNPs. While illumination and pH showed a negative effect. Myco-SNPs were found to be effective against MDR wound pathogens, as evident from the MIC, time killing curve, and post-agent efficiency. This obtained data represented for the first time, the ability of endophytic *A. flavipes* AUMC 15772 under optimum conditions to produce Myco-SNPs that have significant antimicrobial activity against MDR wound pathogens. Remarkably, Myco-SNPs obtained in this work could have a strong application in the pharmaceutical industry as a candidate for wound infection treatment.

## Data Availability

The publication includes a list of all the datasets used in this investigation.

## Abbreviations

AgNO_3_: Silver nitrate
SNPs: Silver nanoparticles
ANOVA: Analysis of variance
CFCF: Cell free cultural filtrate
FTIR: Fourier transform infrared spectroscopy
FWHM: Full width at half maximum
MDR: Multi drug resistant
MH: Mueller Hinton
MIC: Minimum inhibitory concentration
Myco-SNPs: Mycosynthesized silver nanoparticles
OVAT: One variable at a time
PBD: Plackett Burman experimental design
PAE: Post agent efficiency
SAED: Selected area electron diffraction
SDA: Sabrouad dextrose agar
LM: Light microscope
SEM: Scanning electron microscopy
TEM: Transmission electron microscopy
UV–Vis: Ultraviolet–visible spectroscopy
XRD: X-Ray diffraction
